# Epilepsy—Letter from Paris by the Editor of the Southern Med. and Surg. Journal

**Published:** 1847-11

**Authors:** 


*Epilepsy—Letter from Paris by the Editor of the Southern Med. and Surg. Journal.*

Gentlemen—Thanking you, as I have done privately, and now do publicly, for your kind offer to conduct the Journal during my unexpected, and I trust brief absence, I propose to send you a few items of Medical Intelligence by the steamer of the 19th. My sojourn here, however, has been yet so short, that I have but little to communicate.

*Epilepsy.*—You are aware that it is a case of this affection that has brought me here, and which supervened upon premature delivery. I left for the Journal a short article translated from M. Trousseau, containing the wonderful cures he had effected by prolonged warm baths, with a small current of cold water falling at the same time upon the head, in acute diseases within the cranium. I had come to Paris to consult Professors Velpeau, Rostan, and Trousseau, and have already accomplished my object. The latter gentleman, could not, because of previous engagements, meet at the consultation, and therefore made his visit alone. After obtaining a history of the case, which is briefly this—the first attack of convulsions coming on about three weeks after the premature accouchment, then the second about the end of the same interval, when subsequent recurrences varying from four to nine weeks—a permanent pain the head, which has never yet been entirely relieved—failure of all medication, as nitrate of silver, oxide of silver, arsenic, quinine, valerianate of zinc, hydriodate of potash, active purgation, ptyalism, seton to the neck, issues to the arm and leg, cold affusion to the head, diet, narcotics of various kinds, &c., &c., visit to the Madison Springs, Saratoga Springs, travelling, a sea voyage of thirty days—nothing as yet having interrupted the attacks. Prof. Trousseau recommended one medical and one surgical means—the powder of the root or leaves of *belladonna* in small regulated and guarded doses for several months, and *lig tures to the primitive carotids*. He was aware the latter proposition would not be sanctioned by the profession, but he repeatedly said were his own son affected with epilepsy, he would not hesitate a moment to ligate these arteries. We do not know what this disease is, he remarked, and so profound a change in the nutrition of the brain would be produced by closure of the carotids, that I know no means in this affection more available, or which promises as much as their ligation. Prof. Rostan assumed, in consultation with Prof. Velpeau, the management of the case, and promised very kindly to write out in full, directions for it. These I shall not get until too late for this mail. The plan, however, agreed upon by them, consisted in prolonged sedative baths to the skin, hot pediluvia, with cold stream of water to the head, regulation of the bowels, and the powdered root of belladonna.

There being no hereditary tendency in this case, and no permanent external symptom of disease in the brain, they all think, by great care and perseverance in the treatment, that it will be cured. They consider it a severe one, especially on account of its persistence, now sixteen months; and that it is cephalic, and not now dependent on the uterus.

Each of the above named gentlemen utterly refused any compensation for their services in this case.

I may also obtain the opinions of Drs. Marshall Hall, C. J. B. Williams, and Watson, of London, in reference to this case.—*Southern Med. and Surg. Journal.*

